# Nuclear variants of bone morphogenetic proteins

**DOI:** 10.1186/1471-2121-11-20

**Published:** 2010-03-15

**Authors:** Jenny E Felin, Jaime L Mayo, Trina J Loos, J Daniel Jensen, Daniel K Sperry, Stephanie L Gaufin, Christopher A Meinhart, Jennie B Moss, Laura C Bridgewater

**Affiliations:** 1 Department of Microbiology and Molecular Biology, Brigham Young University, Provo, Utah, USA

## Abstract

**Background:**

Bone morphogenetic proteins (BMPs) contribute to many different aspects of development including mesoderm formation, heart development, neurogenesis, skeletal development, and axis formation. They have previously been recognized only as secreted growth factors, but the present study detected Bmp2, Bmp4, and Gdf5/CDMP1 in the nuclei of cultured cells using immunocytochemistry and immunoblotting of nuclear extracts.

**Results:**

In all three proteins, a bipartite nuclear localization signal (NLS) was found to overlap the site at which the proproteins are cleaved to release the mature growth factors from the propeptides. Mutational analyses indicated that the nuclear variants of these three proteins are produced by initiating translation from downstream alternative start codons. The resulting proteins lack N-terminal signal peptides and are therefore translated in the cytoplasm rather than the endoplasmic reticulum, thus avoiding proteolytic processing in the secretory pathway. Instead, the uncleaved proteins (designated nBmp2, nBmp4, and nGdf5) containing the intact NLSs are translocated to the nucleus. Immunostaining of endogenous nBmp2 in cultured cells demonstrated that the amount of nBmp2 as well as its nuclear/cytoplasmic distribution differs between cells that are in M-phase versus other phases of the cell cycle.

**Conclusions:**

The observation that nBmp2 localization varies throughout the cell cycle, as well as the conservation of a nuclear localization mechanism among three different BMP family members, suggests that these novel nuclear variants of BMP family proteins play an important functional role in the cell.

## Background

Bone morphogenetic proteins (BMPs) were first identified nearly 20 years ago as components of a protein extract derived from bone that could direct cartilage and bone formation [1,2]. The BMPs have since been shown to play roles in multiple other developmental pathways [3,4]. For example, Bmp2 provides positional information during axis formation and limb patterning [5,6]. It is expressed in interdigital mesenchyme where apoptosis is occurring, and it induces apoptosis in human myeloma cells [7,8]. In contrast, Bmp2 prevents apoptosis in a chondrocytic cell line and in breast cancer cells [9,10]. In the embryonic lethal Bmp2 null mouse, amnion/chorion development is compromised and the heart is malformed [11,12]. Bmp2 is required for neural crest cell migration, and it promotes neuronal differentiation in neural crest derivatives [12,13]. Bmp2 is also required for embryonic vasculogenesis and promotes tumor angiogenesis [14,15].

BMPs are members of the transforming growth factor β (TGFβ) superfamily. The BMP subfamily, which includes over twenty members, constitutes the largest subfamily in the TGFβ superfamily [16]. In addition to BMPs, the subfamily includes mammalian growth and differentiation factors (GDFs), *decapentaplegic*, 60A and *screw *in *Drosophila*, and *Daf-7 *in *Caenorhabditis elegans *[16]. Like other members of the TGFβ superfamily, BMPs are synthesized as preproproteins and translation is directed to the rough endoplasmic reticulum (ER) by N-terminal signal peptides. While in the secretory pathway, a BMP proprotein is cleaved on the C-terminal side of the proprotein convertase recognition sequence, -R-X-X-R-, to release the C-terminal peptide [17,18]. The C-terminal peptide homodimerizes by disulfide bonding to form the mature secreted growth factor [2,8,19]. Once secreted from the cell, the active BMP dimers signal by binding to heterotetrameric serine/threonine kinase receptor complexes that transduce signals to the nucleus via Smad proteins as well as the mitogen-activated protein kinase (MAPK) pathway [20-23].

The BMPs have previously been recognized only as secreted growth factors. We present evidence herein, however, for the existence of nuclear variants of Bmp2, Bmp4, and Gdf5/CDMP1. We have detected the nuclear variant of Bmp2 (nBmp2) in a variety of different cell types, and we demonstrate that nuclear localization of nBmp2 is directed by a bipartite NLS that overlaps the site of proteolytic cleavage. nBmp2 is translated from an alternative start codon that is located downstream of the ER signal peptide. Without the signal peptide, translation occurs in the cytoplasm and the proprotein avoids the secretory pathway and the proprotein convertases located therein. The bipartite NLS is therefore left intact and directs localization to the nucleus. Examination of other BMP family members revealed that the proteins Bmp4 and Gdf5/CDMP1 are also detectable in the nuclei of cultured cells. Like Bmp2, both contain NLSs that overlap the sites of proprotein processing, and both produce nuclear variants from downstream alternative start codons. The existence of nuclear variants of at least three different BMP family members suggests the conservation of a functional role for these proteins in the nucleus, representing a novel mechanism of BMP function.

## Results

### Immunostaining reveals nuclear Bmp2

**Figure 1:**
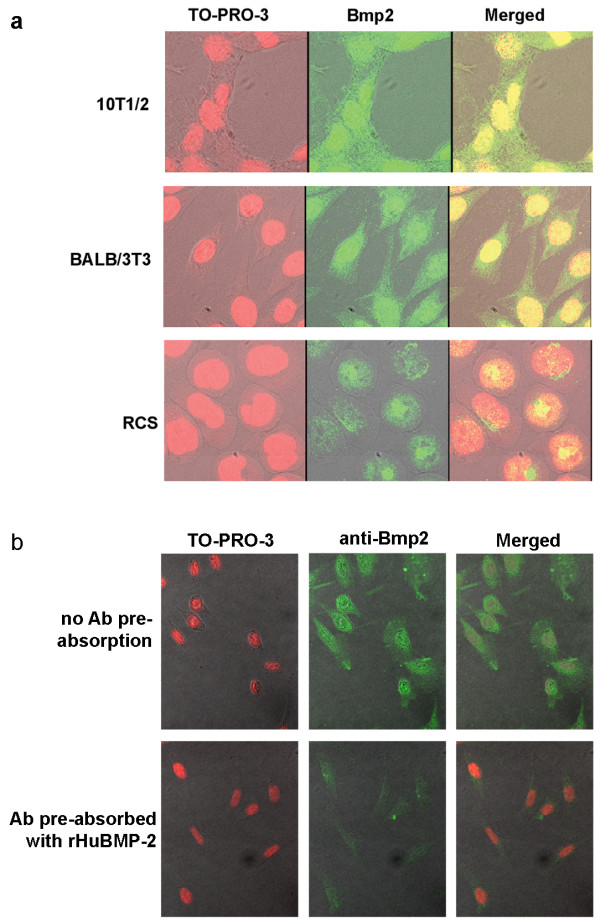
**Endogenous Bmp2 is detectable in the nuclei of three cultured cell lines**. **(a) **Non-transfected 10T1/2 mesenchymal cells, BALB/3T3 fibroblasts, and RCS cells were cultured on slides and immunostained using an anti-Bmp2 antibody (green). Nuclei were stained with TO-PRO-3 (red), and cells were examined by laser confocal microscopy. **(b) **Antibody specificity was verified by preabsorbing anti-Bmp2 antibody with recombinant human BMP-2 before immunostaining.

Using DNA affinity chromatography followed by mass spectrometry, we recently observed fragments of the Bmp2 proprotein in nuclear extracts from rat chondrosarcoma (RCS) cells. Suspecting cytoplasmic contamination, we performed immunofluorescent staining of three cell lines: 10T1/2 mesenchymal cells, BALB/3T3 fibroblasts, and RCS cells, using primary antibodies against Bmp2 ([N-14]:sc-6895, Santa Cruz Biotechnology). Cells were imaged using an Olympus IX81 laser confocal microscope to allow imaging of cellular cross-sections in order to distinguish staining that was at or immediately outside of the nuclear envelope from staining that was truly nuclear. All three cell lines showed true nuclear localization of Bmp2 (Figure 1a). Antibody specificity was confirmed using side-by-side comparison of BALB/3T3 cells stained with antibody that was or was not pre-absorbed with recombinant human BMP-2 (GenScript, Piscataway, NJ) (Figure 1b). Pre-absorption inhibited antibody staining, including nuclear staining, confirming that the antibody used is specific for Bmp2.

### Identification of the nuclear localization signal

**Figure 2:**
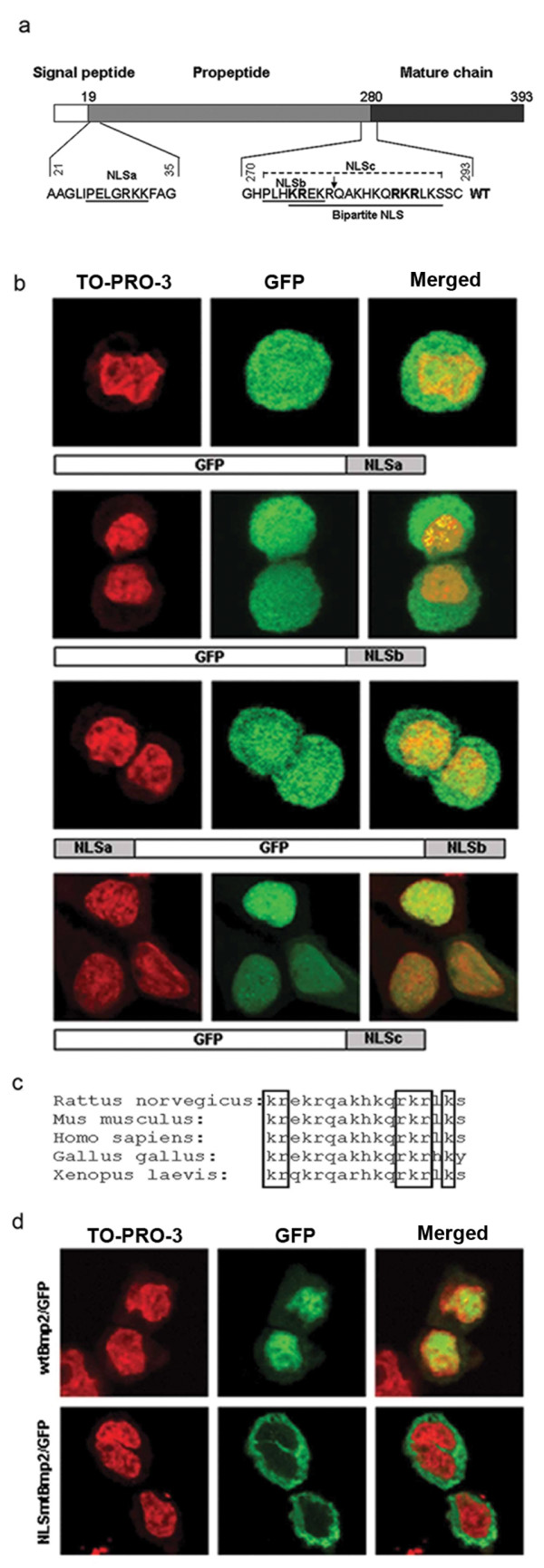
**Bmp2 contains a functional bipartite NLS that overlaps the site of proteolytic processing**. **(a) **Map of the Bmp2 preproprotein showing the signal peptide, propeptide, and mature chain. The amino acid sequence and location of each predicted NLS is shown, and the site of proteolytic cleavage is marked by an arrow. **(b) **Four GFP/NLS fusion genes were constructed as shown to test the ability of each predicted NLS to direct GFP to the nucleus. These expression vectors were transfected into RCS cells, and GFP localization (green) was visualized using laser confocal microscopy. Nuclei were stained with TO-PRO-3 (red). Only the bipartite NLSc directed strong nuclear localization. **(c) **Alignment of Bmp2 bipartite NLSc sequences from five different species. The six basic amino acids that characterize this sequence as a bipartite NLS are boxed. **(d) **To determine whether the bipartite NLSc is functional within Bmp2, GFP was fused to the C-terminus of the full-length Bmp2 preproprotein (wtBmp2/GFP). In a parallel fusion construct, the bipartite NLSc was mutated (NLSmtBmp2/GFP). These plasmids were transfected into RCS cells, and GFP localization was visualized using laser confocal microscopy. Nuclei were stained with TO-PRO-3 (red).

The PSORT II program http://psort.ims.u-tokyo.ac.jp/ was used to predict potential nuclear localization signals (NLS) in the rat Bmp2 proprotein amino acid sequence. Three candidate NLSs were identified. The first, PELGRKK (named NLSa), is positioned at amino acids 26-32, almost immediately following the signal peptide. The second, PLHKREK (named NLSb), is located at amino acids 272-278, at the C-terminus of the propeptide. The third, KREKRQAKHKQRKRLKS (named NLSc), is a bipartite nuclear localization signal situated at amino acids 275-291, which overlaps both NLSb and the site of proteolytic cleavage (Figure 2a).

To determine whether these potential NLSs were capable of directing nuclear localization, we fused each one to the C-terminal end of green fluorescent protein (GFP). The fusion constructs were transiently transfected into RCS cells, and cells were stained with the DNA-specific stain TO-PRO-3 iodide to highlight nuclei. Transfected cells were examined using an Olympus IX81 laser confocal microscope. Neither NLSa nor NLSb produced nuclear localization of GFP (Figure 2b). Rather, GFP (a 26 kDa protein) fused to the short NLSs was distributed throughout both the cytoplasm and the nucleus as would be expected for a protein that is small enough (<30-40 kD) to diffuse through nuclear pores [24,25].

Because the two short putative NLSs bracket the free propeptide that would be released upon proteolytic cleavage of the Bmp2 proprotein, we investigated whether the two NLSs might cooperate to transport the free propeptide to the nucleus. We built a construct in which the N-terminal putative NLSa was fused to the N-terminus of GFP and the C-terminal putative NLSb was fused to the C-terminus of GFP. Transient transfection of this construct into RCS cells revealed that even together, the two short NLSs failed to produce nuclear localization of GFP (Figure 2b).

Finally, we fused the putative bipartite NLSc, also containing NLSb (PLHKREKRQAKHKQRKRLKS), to the C-terminus of GFP. Transient transfection of this expression plasmid into RCS cells produced clear nuclear localization of GFP, indicating that the predicted bipartite NLSc is indeed functional (Figure 2b). Comparison of rat Bmp2 with the amino acid sequences of mouse, human, chicken, and frog Bmp2 showed that the critical basic amino acids in the bipartite NLS are 100% conserved between these species (Figure 2c).

To determine whether the bipartite NLS was functional in the context of full-length Bmp2, we built a fusion construct designed to express the entire Bmp2 proprotein, including the signal peptide, with GFP fused to its C-terminus. This wtBmp2/GFP plasmid was transiently transfected into RCS cells, and results were analyzed by cell counting using laser confocal microscopy. Of the cells expressing GFP, 21 ± 3% showed nuclear localization of the wtBmp2/GFP fusion protein (Figure 2d). The remainder of the GFP-expressing cells showed Bmp2/GFP either evenly distributed in the nucleus and cytoplasm, or predominantly localized to the cytoplasm. A control plasmid expressing only GFP produced diffuse GFP localization in both the cytoplasm and nucleus of all transfected cells as previously observed by others [25], verifying that the Bmp2 portion of the fusion protein is required for nuclear localization (data not shown).

To confirm that the bipartite NLS was necessary for nuclear localization of Bmp2, a targeted mutation of five amino acids within the bipartite NLS in the Bmp2/GFP fusion construct (**KR**EKRQAKHKQ**RKR**LKS changed to **AA**EKRQAKHKQ**AAA**LKS) was generated (NLSmtBmp2/GFP). When transfected into RCS cells, this construct produced 0% nuclear localization of Bmp2/GFP, confirming that the bipartite NLS that overlaps the site of proteolytic cleavage was essential for nuclear localization (p < 0.001), and that no other sequence elements could compensate for the loss of the bipartite signal (Figure 2d). This result implied that the nuclear version of Bmp2 is uncleaved, as cleavage would split and thus destroy the bipartite NLS.

**Figure 3:**
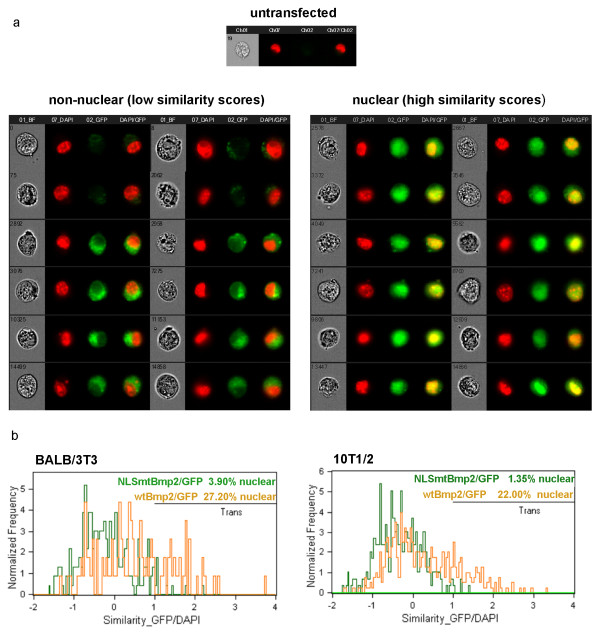
**The bipartite NLS directs nuclear localization of Bmp2**. BALB/3T3 and 10T1/2 cells were transfected with wtBmp2/GFP or NLSmtBmp2/GFP, stained with DAPI (red in these images), and analyzed by ImageStream^® ^imaging flow cytometry. **(a) **Representative images of cells expressing no GFP (top panel), and of cells with low (non-nuclear localization) and high (nuclear localization) similarity scores. **(b) **Overlay histograms comparing similarity scores from wtBmp2/GFP and NLSmtBmp2/GFP in BALB/3T3 and 10T1/2 cells. The percentage of cells with nuclear GFP was defined by gating the events with high similarity scores as shown by the horizontal lines in the top right of each histogram. Percentages of cells with nuclear GFP are given (upper right).

The nuclear localization of the wtBmp2/GFP fusion protein and the necessity of the NLS was further confirmed and quantified by transfecting wtBmp2/GFP and NLSmtBmp2/GFP plasmids into 10T1/2 and BALB3T3 cells, staining with DAPI, and analyzing GFP localization using an ImageStream^® ^multispectral quantitative imaging flow cytometer. The ImageStream^® ^sorted for cells that expressed GFP and then imaged each of these cells individually. Representative images of cells with non-nuclear and nuclear localization of the GFP fusion protein are shown in Figure 3a. An untransfected cell is also shown (Figure 3a, top panel).

The percent of GFP-expressing cells in each sample that displayed nuclear localization of the GFP fusion proteins was assessed by measuring the 'Similarity' of the GFP and DAPI images on a per cell basis. The Similarity score is a log-transformed Pearson's correlation coefficient of the pixel values of the DAPI and GFP images [26]. If GFP is localized to the nucleus, the two images are similar and have large positive values. Similarity GFP/DAPI overlay histograms for wtBmp2/GFP (yellow) and NLSmtBmp2/GFP (green) in each cell line are shown (Figure 3b). The percentage of cells with nuclear GFP was defined by gating the events with high similarity scores at the points indicated by the horizontal black line in the upper right of each histogram. BALB/3T3 and 10T1/2 cells showed 27.2% and 22.0% nuclear localization of the wtBmp2/GFP fusion protein, respectively. Mutation of the NLS reduced nuclear localization to 3.9% and 1.4%, demonstrating once more that the bipartite NLS directs nuclear localization of Bmp2 (Figure 3b).

### Preventing proprotein cleavage does not increase levels of nBmp2

**Figure 4:**
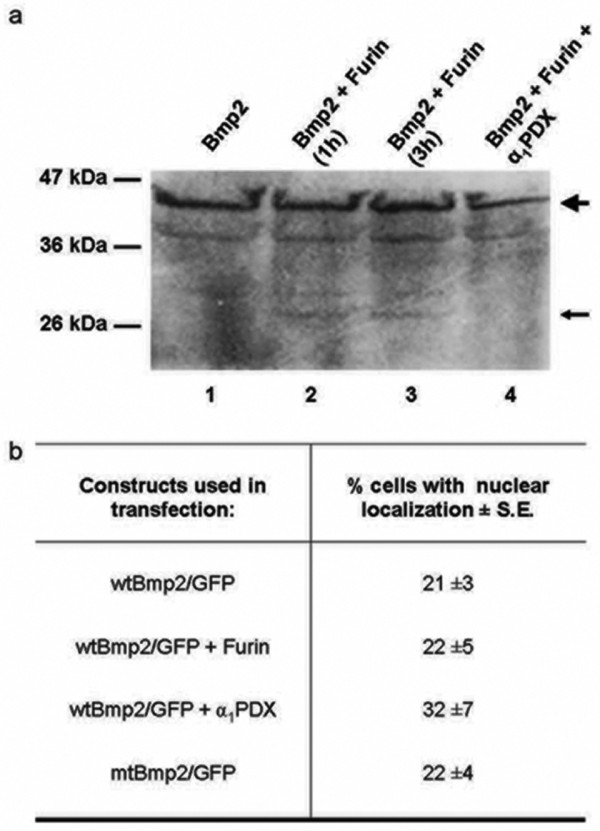
**Inhibition of Bmp2 proprotein processing does not increase nuclear localization**. **(a) ***In vitro *synthesis of radiolabeled Bmp2 preproprotein produced a 43 kDa protein (lane 1, large arrow). Incubation of this protein with recombinant furin for 1 or 3 hours generated a new protein band at 31 kDa, the predicted size of the free Bmp2 propeptide following proteolytic cleavage (lanes 2 and 3, small arrow). Preincubation of the furin with α_1_PDX, a serine protease inhibitor that blocks furin activity, prevented the formation of this band (lane 4). **(b) **Furin and α_1_PDX expression plasmids were each cotransfected with the wtBmp2/GFP fusion plasmid into RCS cells. Increasing furin expression did not significantly decrease nuclear localization of Bmp2/GFP, nor did inhibiting furin activity with α_1_PDX significantly increase nuclear localization. Mutation of the Bmp2 cleavage site to make it unrecognizable by furin or any related proprotein convertase (mtBmp2/GFP) also failed to significantly increase nuclear localization of Bmp2/GFP.

We examined the possibility that uncleaved nuclear Bmp2 could be produced by inhibition of the proprotein convertase responsible for its cleavage. The cleavage site of Bmp2, R-E-K-R-^↓^, is a consensus site for furin as well as for several related members of the proprotein convertase family, and furin cleaves Bmp4, the BMP family member most closely related to Bmp2 [27-29]. To determine whether furin can cleave Bmp2, we used an *in vitro *protein cleavage assay. This showed that furin can cleave Bmp2 (Figure 4a, lanes 2 and 3) and that cleavage can be prevented by α_1_-PDX, a serine protease inhibitor (serpin) that is highly selective in its inhibition of furin (Figure 4a, lane 4) [30-32].

To examine whether the production of nuclear Bmp2 can be increased *in vivo *by inhibiting proteolytic processing of the Bmp2 proprotein by furin, we utilized the α_1_-PDX expression plasmid α1-Portland (provided by Dr. Gary Thomas at the Oregon Health Sciences University in Portland, Oregon). Cotransfection of this plasmid with the wtBmp2/GFP fusion plasmid into RCS cells did not produce a statistically significant increase in the percentage of transfected cells showing nuclear localization (p = 0.145). Likewise, cotransfection of the Bmp2/GFP fusion plasmid with a furin expression plasmid did not decrease the percentage of transfected cells showing nuclear localization (p = 0.810) (Figure 4b).

Because it is possible that another proprotein convertase besides furin is responsible for the proteolytic cleavage of Bmp2, we constructed a cleavage mutant of Bmp2/GFP with a disrupted consensus cleavage site but with the essential amino acids in the bipartite NLS intact, designated mtBmp2/GFP (KREK**R**QAKHKQRKRLKS was changed to KREK**G**QAKHKQRKRLKS). This mutant cannot be proteolytically processed by any member of the proprotein convertase family, because it lacks the essential arginine in the fourth position of the minimal proprotein convertase recognition sequence (R-X-K/R-R) leaving the NLS always intact [33]. This mutation, however, also failed to alter the percentage of transfected cells showing nuclear localization (p = 0.652), suggesting that regulation of the proprotein convertase(s) involved in proteolytic processing of Bmp2 is probably not the mechanism by which the uncleaved nuclear variant of Bmp2 is produced (Figure 4b).

### nBmp2 translation is initiated from a downstream alternative start codon

**Figure 5:**
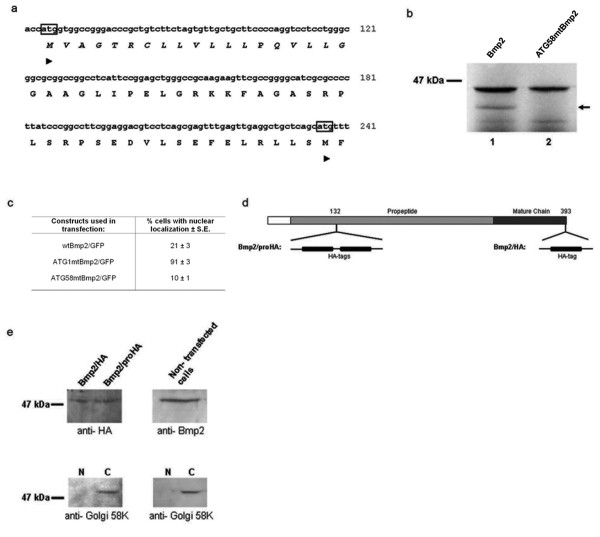
**Translation of Bmp2 from an alternative start codon downstream of the signal peptide produces the uncleaved nuclear variant of Bmp2**. **(a) **The N-terminus of the rat Bmp2 protein with corresponding DNA sequence is shown. The conventional (codon 1) and predicted alternative (codon 58) start codons are marked (arrowheads), and the signal peptide is shown in italics. **(b) ***In vitro *synthesis of Bmp2 produced the expected 42 kDa protein and some lower molecular weight proteins (lane 1). Mutation of codon 58 eliminated one of the smaller proteins, indicating that it was initiated at codon 58 (arrow). **(c) **Bmp2/GFP fusion constructs containing substitutions in either the conventional start codon 1 (ATG1 mtBmp2/GFP) or the alternative start codon 58 (ATG58 mtBmp2/GFP), were transfected into cells, and the percentage of transfected cells showing nuclear localization was quantified. **(d) **HA tags were inserted into the propeptide or fused to the C-terminus of Bmp2 as shown. **(e) **The HA tagged expression vectors were transfected into RCS cells, and nuclear extracts were analyzed by western blotting. Both vectors produced an HA-tagged nuclear protein of ~50 kDa (top left panel). When nuclear extracts from non-transfected RCS cells were analyzed by western blotting using an anti-Bmp2 antibody, a ~50 kDa endogenous nuclear protein was labeled (top right panel--note that this panel contains only one wide lane). Nuclear (N) and cytoplasmic (C) extracts were probed using a Golgi-specific antibody to verify that the nuclear extracts were not contaminated with cytoplasmic proteins (bottom panels).

We also considered the possibility that uncleaved Bmp2 could be produced by initiating translation at a downstream alternative start codon, which would eliminate the signal peptide. Such a protein would not be directed to the ER and the secretory pathway, and would thus avoid proteolytic processing. We examined the nucleotide sequence of rat Bmp2 mRNA and found, downstream of the conventional ATG initiation codon, an in-frame ATG at codon 58 that was surrounded by a partial Kozak sequence (Figure 5a) [34]. The NetStart 1.0 Prediction program http://www.cbs.dtu.dk/services/NetStart/ indicated that codon 58 is the second most likely translational start site in the Bmp2 transcript with a score of 0.724, compared to 0.848 for the conventional initiator codon 1. The mouse, human, chicken, and frog Bmp2 sequences were also all predicted by the NetStart 1.0 program to contain strong alternative start sites at codons 58, 59, or 60.

To test the hypothesis that codon 58 is used as an alternative start codon, Bmp2 was transcribed and translated *in vitro *in the presence of ^35^S-methionine. When the radiolabeled protein products were separated by SDS-PAGE, the major expected band of 42 kDa appeared as well as a minor band of approximately 38 kDa, consistent with the predicted size of a protein initiated at codon 58 (Figure 5b, lane 1). Mutation of codon 58 to a non-initiator AAG codon eliminated the 38-kDa band, indicating that this protein was indeed produced by initiating translation at codon 58 (Figure 5b, lane 2).

To determine whether codon 58 can also function as an alternative start site *in vivo*, we generated Bmp2/GFP fusion constructs containing substitution mutations in either the conventional start codon 1 or in codon 58. These constructs were transfected into RCS cells, and subcellular localization was visualized by fluorescent laser confocal microscopy. The wtBmp2/GFP fusion construct produced nuclear localization in 21 ± 3% of transfected cells. Mutation of the conventional start codon to compel utilization of the downstream alternative start codon dramatically increased nuclear localization to 91 ± 3% of transfected cells. In contrast, mutation of the alternative start codon 58 reduced nuclear localization to only 10 ± 1%, indicating that codon 58 is utilized to produce the nuclear variant of Bmp2 (nBmp2) *in vivo *(Figure 5c).

To verify that nBmp2 is an uncleaved variant containing part of the propeptide and the bipartite NLS, two copies of a hemaglutinin (HA) tag were inserted into the full length Bmp2 cDNA between amino acids 132 and 133 in the propeptide region (Bmp2/proHA). (Two copies of the tag were utilized because the anti-HA antibody was unable to recognize a single copy of the tag embedded in the propeptide.) In a second construct, a single HA tag was inserted at the C-terminus of Bmp2 (Bmp2/HA) (Figure 5d). These expression vectors containing HA-tagged Bmp2 were transfected into RCS cells, and nuclear and cytoplasmic extracts were prepared. To verify that the nuclear extract was free from cytoplasmic contamination, the nuclear and cytoplasmic fractions were subjected to immunoblotting using a Golgi-specific antibody (anti-Golgi 58K) (bottom two panels, Figure 5e). Immunoblotting with an anti-HA antibody revealed a protein of ~50 kDa in the nuclear extract, regardless of whether the HA tag was embedded in the propeptide or placed at the C-terminus of Bmp2, indicating that the nuclear variant begins at a point somewhere between the signal peptide and amino acid 133, and extends to the C-terminus of the conventional secreted growth factor. The nuclear variant of Bmp2 is clearly not cleaved in the middle of the bipartite NLS (top left panel of Figure 5e).

In order to determine whether cultured cells contain an endogenous Bmp2 protein that is localized to the nucleus and matches the molecular weight of our ectopically expressed HA-tagged Bmp2, nuclear extracts were prepared from untransfected RCS cells and examined by immunoblotting using an anti-Bmp2 antibody. Once again, a protein of ~50 kDa was detected in the nuclear extract, indicating that the translation and localization of ectopically expressed nBmp2 accurately reflects the translation and localization of endogenous nBmp2 (top right panel, Figure 5e). (Note that the wells used on the gel shown in the top right panel of Figure 5e were twice as wide as those shown in the other three panels, so the top right panel shows one lane while the other three panels contain two lanes each.) In addition to supporting our proposed model for nBmp2 translation and localization, these immunoblotting results also confirm the Figure 1 immunofluorescence results showing endogenous nBmp2 in the nuclei of untransfected cells.

The calculated molecular weight of nBmp2 translated from the predicted alternative start codon at amino acid 58 is 38 kDa. This is consistent with the size of the Bmp2 protein produced *in vitro *where post-translational modifications do not occur (Figure 5b). Bmp2, however, is post-translationally modified *in vivo*. This causes the full-length preproprotein, which has a predicted molecular weight of 42 kDa, to migrate at about 66 kDa on SDS-PAGE [2,35]. It is probable, then, that post-translational modifications also account for the discrepancy between the calculated size of nBmp2 (38 kDa) and its observed electrophoretic mobility (50 kDa).

### Nuclear localization of nBmp2 varies throughout the cell cycle

**Figure 6:**
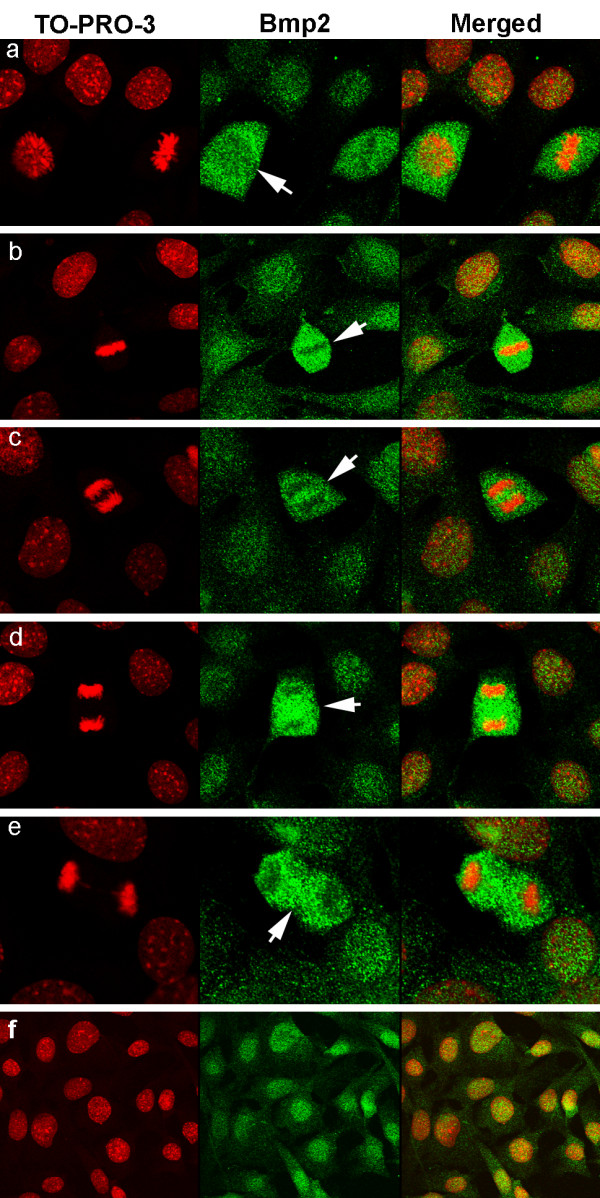
**The intensity of staining and nuclear localization of nBmp2 differ between M-phase and the other phases of the cell cycle**. 10T1/2 cells were cultured on slides for 24 hrs in the absence of serum, and then serum was replaced at time zero. Cells were immunostained using an anti-Bmp2 antibody (green) to visualize nBmp2 in cells at different stages of the cell cycle. Nuclei were stained with TO-PRO-3 (red), and cells were examined by laser confocal microscopy. Panels a-e show mitotic cells imaged at the 4 hr. time point. **(a) **A cell in late prophase (arrow) shows more nBmp2 staining than surrounding non-mitotic cells, with the least intense staining in the region of chromosome condensation. A metaphase cell is also visible in this frame. Cells in metaphase **(b)**, anaphase **(c)**, and telophase **(d) **(arrows) show more intense Bmp2 staining than surrounding non-mitotic cells, and staining is reduced where condensed chromosomes are located. **(f) **Non-mitotic cells imaged at the 8 hr. time point show nuclear localization of nBmp2.

The observation that only about 20% of the cells expressing wtBmp2/GFP display nuclear localization of GFP at any given moment suggested that the nuclear localization of nBmp2 may vary throughout the cell cycle. To address this question, we serum-starved 10T1/2 cells for 24 hours and then returned serum to the culture medium so many cells would enter mitosis simultaneously. At various time points after serum replacement, cells were stained by immunofluorescence using primary antibodies against endogenous Bmp2 ([N-14]:sc-6895, Santa Cruz Biotechnology) and imaged using an Olympus IX81 laser confocal microscope. The four-hour time point yielded many mitotic cells, including cells in prophase, metaphase, anaphase, telophase, and cytokinesis. Cells that were not undergoing mitosis showed predominantly nuclear localization of Bmp2/GFP (Figure 6f). In contrast, cells in late prophase, when nuclear envelope breakdown occurs, showed intense Bmp2/GFP staining throughout the entire cell except in the location of chromosome condensation (Figure 6a). Cells in metaphase, anaphase, and telophase also showed more intense Bmp2/GFP staining than surrounding cells, but staining was excluded from the site where condensed chromosomes were located (Figure 6b-d). In cells undergoing cytokinesis, when chromosomes were decondensing and nuclear membranes reforming, Bmp2/GFP staining was cytoplasmic (Figure 6e), suggesting that nuclear translocation of nBmp2 is necessary after each cell division to re-establish the pattern of nuclear localization observed in non-mitotic cells (Figure 6f).

### nBmp4 and nGdf5 are produced by similar mechanisms

**Figure 7:**
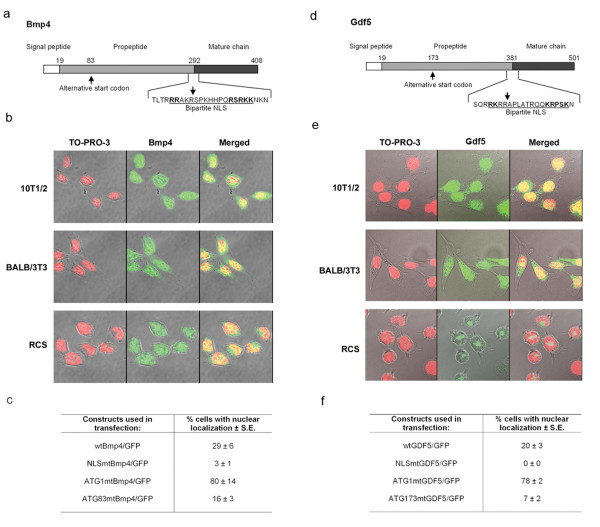
**Nuclear variants of Bmp4 and Gdf5 are also translated from downstream alternative start codons and contain NLSs that overlap the sites of proprotein processing**. **(a) **Map of the Bmp4 preproprotein showing the signal peptide, propeptide, and mature chain. The amino acid sequence and location of the bipartite NLS are shown, and the alternative start codon and site of proteolytic cleavage are marked. **(b) **Endogenous Bmp4 is detectable in the nuclei of three cultured cell lines. Non-transfected 10T1/2, BALB/3T3, and RCS cells were cultured on slides and immunostained using an anti-Bmp4 antibody (green). Nuclei were stained with TO-PRO-3 (red), and cells were examined by laser confocal microscopy. **(c) **Bmp4/GFP fusion constructs containing targeted mutations in the NLS, the conventional start codon 1, or the alternative start codon 83, were transfected into RCS cells to examine the effects of these mutations on nuclear localization of Bmp4. **(d) **Map of the Gdf5 preproprotein showing the signal peptide, propeptide, and mature chain. The amino acid sequence and location of the bipartite NLS are shown, and the alternative start codon and site of proteolytic cleavage are marked. **(e) **Endogenous Gdf5 is detectable in the nuclei of two of the three cultured cell lines shown. Non-transfected 10T1/2, BALB/3T3, and RCS cells were cultured on slides and immunostained using an anti-Gdf5 antibody (green). Nuclei were stained with TO-PRO-3 (red), and cells were examined by laser confocal microscopy. **(f) **GDF5/GFP fusion constructs containing targeted mutations in the NLS, the conventional start codon 1, or the alternative start codon 173, were transfected into RCS cells to examine the effects of these mutations on nuclear localization of Gdf5.

To determine whether the existence of nuclear variants might be conserved among other BMP family members, we utilized the PSORT II program http://psort.ims.u-tokyo.ac.jp/ to search their amino acid sequences for putative NLSs. Two proteins, Bmp4 and Gdf5, were selected for further study because each contained a predicted bipartite NLS overlapping the site of proprotein processing, as in Bmp2 (Figure 7a and 7d). When fused to GFP, both of these predicted NLSs were found capable of directing translocation of GFP to the nucleus in RCS cells (data not shown). Immunofluorescent staining was performed on cultured 10T1/2, BALB/3T3, and RCS cells to see if endogenous Bmp4 and Gdf5 could be detected in the nuclei. Using primary antibodies against Bmp4 (JM-5674-100, MBL International), we found that nuclear Bmp4 was detectable in all three cell lines (Figure 7b). Using primary antibodies against Gdf5 ((N-17):sc-6901, Santa Cruz Biotechnology), we found that Gdf5 was detectable in the nuclei of 10T1/2 and BALB/3T3 cells, but was not localized to the nucleus in RCS cells (Figure 7e).

To determine whether the bipartite NLSs were necessary for the nuclear localization of Bmp4 and Gdf5, GFP was fused to the C-terminus of each full-length protein. When transfected into RCS cells, the wtBmp4/GFP fusion construct showed nuclear localization in 29 ± 6% of transfected cells (Figure 7c) and the wtGDF5/GFP fusion construct showed nuclear localization in 20 ± 3% of transfected cells by manual counting on an Olympus IX81 laser confocal microscope (Figure 7f). Targeted mutation of the bipartite NLS in Bmp4 (**RR**AKRSPKHHPQRS**RKK **was changed to **AA**AKRSPKHHPQRS**AAV**) markedly reduced nuclear localization (p < 0.001) (NLSmtBmp4/GFP, Figure 7c). Targeted mutation of the bipartite NLS in Gdf5 (**RK**RRAPLATRQQ**KR**PSK changed to **AA**RRAPLATRQQ**AA**PSK) completely eliminated nuclear localization (p < 0.001) (NLSmutGDF5/GFP, Figure 7f), demonstrating that the bipartite NLSs overlapping the sites of proprotein processing are necessary for nuclear localization of both Bmp4 and Gdf5.

Because nBmp2 is translated from a downstream alternative start codon, Bmp4 and Gdf5 were examined using the NetStart 1.0 Prediction program http://www.cbs.dtu.dk/services/NetStart/ to identify possible alternative start codons. Bmp4 contained a predicted alternative start site at codon 83 (Figure 7a). When the conventional start site at codon 1 was mutated (ATG changed to AAG) in the context of the Bmp4/GFP fusion protein to force utilization of downstream alternative start codons (ATG1 mtBmp4/GFP), nuclear localization increased to 80% of transfected cells (p = 0.035) (Figure 7c). Targeted mutation of codon 83 (ATG83 mtBmp4/GFP), in contrast, reduced nuclear localization by approximately one-half to 16% (p = 0.010) (Figure 7c), indicating that codon 83 can indeed be used as a start site for translation of the nuclear variant of Bmp4 (nBmp4).

When the conventional start site at codon 1 in Gdf5 was mutated (ATG changed to AAG) in the context of the GDF5/GFP fusion construct (ATG1 mtGDF5/GFP) to force utilization of downstream alternative start codons, nuclear localization increased to 78% of transfected cells (p < 0.001), indicating that the nuclear variant of Gdf5 is also translated from one or more downstream alternative start codons (Figure 7f). Mutation of a possible CTG alternative start site (CTG changed to AAG) at codon 53 did not change the percent of transfected cells showing nuclear localization of GDG5/GFP, suggesting that this site does not serve as an alternative start codon (data not shown). The first in-frame ATG after codon 1 is found at codon 173. When this site was mutated (ATG changed to AAG), nuclear localization was reduced to 7% of transfected cells (p = 0.012) (Figure 7f), indicating that codon 173 can serve as an alternative start codon for the translation of nuclear Gdf5 (nGdf5).

## Discussion

The results presented here demonstrate that translation that begins downstream of the ER signal peptide drives nuclear localization of Bmp2, Bmp4, and Gdf5. In each case, elimination of the signal peptide prevents translation of the nascent polypeptide into the ER and thereby prevents transit through the secretory pathway, which in turn prevents contact with the Golgi-localized proprotein convertases that would otherwise cleave the proprotein and destroy its NLS. These findings indicate that nuclear Bmp2, Bmp4, and Gdf5 proteins translated from alternative downstream start codons cannot function in their traditional role as ligands binding to cell surface receptors, because they cannot enter the secretory pathway. Likewise, processed and secreted Bmp2, Bmp4, and Gdf5 would not be likely to enter the nucleus after binding to cell surface receptors and being internalized, as their NLSs are destroyed by proprotein processing. This mechanism for producing nuclear variants of BMP family proteins stands in contrast to several other growth factors including EGF family members, IFNγ, FGF family members, prolactin, and growth hormone [36,37]. In these examples, growth factors are secreted from the cell, bind to plasma membrane receptors, and are internalized prior to translocation of the ligand and/or its receptor to the nucleus.

The nuclear localization of growth factor variants that avoid the secretory pathway is not unprecedented. A nuclear form of parathyroid hormone-related peptide (PTHrP) can be generated by translation from an alternative start site downstream of the conventional initiator ATG, producing a protein with a truncated signal peptide much like nBmp2, nBmp4, and nGdf5. Loss of the signal peptide enables PTHrP to bypass the ER and secretory pathway, and an embedded NLS then interacts with importin β1 to direct PTHrP to the nucleus [38,39]. Another example of nuclear localization due to utilization of an alternative start codon is found in the basic fibroblast growth factor (bFGF). In one form of this protein, nuclear localization is determined by translational initiation at an upstream alternative start site. The utilization of an upstream CUG start codon produces a variant protein with an extended amino terminus containing an NLS that directs nuclear localization [40]. In the case of fibroblast growth factor 3 (FGF3), translation can initiate at a CUG codon that is 87 nucleotides upstream of the first AUG in the protein-coding frame. These 87 nucleotides code for two NLS signals and a hydrophobic secretory signal, and the balance between nuclear localization and secretion of this protein variant is determined by the competing signals [41,42]. These examples demonstrated a precedent for altering the subcellular localization of proteins by initiating translation from alternative start sites, and they show that nuclear localization of growth factors can occur without prior secretion of the protein from the cell [43].

The novel location of the bipartite NLS in Bmp2, overlapping the site of proprotein processing, initially led us to consider a different mechanism of nuclear localization. We examined whether inhibition of proprotein processing might leave the NLS intact and thus lead to nuclear localization of Bmp2. This hypothesis was considered because regulation of furin activity has been shown to affect the activity of furin substrates in other cases. For example, the pro-β-NGF protein is a neurotropin that has opposing activities depending on whether or not it is cleaved by furin. Cleaved β-NGF promotes cell survival, whereas uncleaved β-NGF promotes apoptosis of neurons [44]. Furin is also responsible for cleavage of the transmembrane receptor Notch. Cleavage results in the release of the Notch intracellular domain, which goes to the nucleus and activates genes involved in development and differentiation [45]. Uncleaved Notch, in contrast, inhibits cell differentiation [46]. The experiments presented here, however, do not support a role for furin modulation in regulating the localization of Bmp2.

Instead, this work has demonstrated that nBmp2, nBmp4, and nGdf5 are all produced by initiating translation from a location downstream of the signal peptide. In each protein, an alternative start codon was identified, the mutation of which reduced nuclear localization by approximately 50%. These codons were located at amino acid positions 58, 83, and 173 in Bmp2, Bmp4 and Gdf5, respectively. Interestingly, these different start sites produce nuclear variants that are 336, 326, and 329 amino acids long for nBmp2, nBmp4, and nGdf5, respectively. The differences in the lengths of the three proproteins, therefore, are almost entirely accounted for by differences upstream of the alternative start codons, suggesting that selective pressure has played a role in maintained the lengths of the nuclear variants.

The observation that mutation of the alternative start codons reduced but did not eliminate synthesis of the nuclear variants of each protein suggests that, at least in ectopically expressed fusion constructs, other sites can serve as start codons for synthesis of the nuclear variants when the primary alternative start sites are eliminated. Indeed, the NetStart 1.0 program predicted several weaker alternative start codons in each propeptide coding region. It is not clear, however, whether usage of any other alternative start codons ever occurs *in vivo*.

The observation that only 20-30% of cells that expressed the three wild type BMP/GFP fusion constructs showed nuclear localization of the fusion proteins suggested that translational start site selection and/or nuclear translocation might be regulated, perhaps in association with the cell cycle. Indeed, immunofluorescence staining of endogenous Bmp2 in cells undergoing mitosis demonstrated that cells entering M-phase of the cell cycle display the most intense staining, which might reflect increased utilization of the alternative start codon during the G_2_/M phase of the cell cycle. A similar pattern of subcellular localization throughout the cell cycle has been reported for PTHrP, and it has been suggested that this pattern "supports a role for PTHrP in cell division" [47]. Dissolution of the nuclear envelope seemed to allow nBmp2 to spread throughout the cell, either by diffusion or active transport, and when the nuclear envelope reassembled during cytokinesis, nBmp2 was no longer preferentially localized to the nucleus. These observations suggest that new nuclear translocation of nBmp2 is required to re-establish nuclear localization every time a cell completes M-phase of the cell cycle. Additional experiments will be required to determine whether the same nBmp2 molecules can be re-transported to the nucleus, or whether de novo protein synthesis is required after each cell division. Likewise, additional experiments are needed to explore whether nBmp2 plays any role in cell division.

## Conclusions

In summary, the experiments presented here have demonstrated by immunofluorescence staining that endogenous Bmp2 is detectable in the nuclei of three different cultured cell lines. GFP fusion constructs showed that the bipartite NLS overlapping the site of proteolytic cleavage is essential for nuclear localization of Bmp2, and that the nuclear variant of Bmp2 is produced from a downstream alternative start codon. These results are further supported by immunoblots demonstrating the presence of endogenous nBmp2 in nuclear extracts and showing that the electrophoretic mobility of endogenous nBmp2 is the same as that of ectopically expressed, HA-tagged nBmp2. Nuclear localization of nBmp2 was shown to vary as cells progressed through the cell cycle, consistent with the observation that only about 20% of transfected cells showed nuclear localization of ectopic wtBmp2/GFP at any given time. The Bmp2 data described here is further bolstered by the demonstration that BMP family members Bmp4 and Gdf5 are also detectable in the nucleus by immunocytochemistry and are synthesized and localized to the nucleus by similar means. Together, these results indicate that Bmp2, Bmp4, and Gdf5 can be alternatively translated as secreted growth factors or as nuclear proteins.

Conservation of nuclear variants of three different BMP family members, and conservation of the mechanism by which their nuclear localization occurs, suggests a conserved functional role for these three proteins in the nucleus. The observation that nBmp2 localization differs at different stages of the cell cycle also suggests a functional role for this novel protein. Our earliest observation of nBmp2 among nuclear proteins that had been purified by DNA affinity chromatography suggests that nBmp2 may bind DNA, perhaps to regulate transcription. Computational analysis, however, shows no predicted DNA-binding domain in nBmp2. Furthermore, electrophoretic mobility shift assays have so far failed to show direct binding of nBmp2 to DNA. It remains possible that nBmp2 interacts indirectly with DNA as part of a protein complex, and this possibility is currently being explored by examining the array of proteins with which nBmp2 interacts. We have also used targeted mutagenesis to produce a mouse in which nBmp2 cannot be translocated to the nucleus. Analysis of this mouse's phenotype is currently underway and is just beginning to yield interesting insights into the functional role of nBmp2.

## Methods

### Construction of plasmids and mutagenesis

The pCMV/GFP-NLSa, pCMV/GFP-NLSb and pCMV/GFP-NLSc constructs were generated by annealing complementary oligonucleotides encoding PELGRKK, PLHKREK and PLHKREKRQAKHKQRKRLKS respectively with additional nucleotides to create *Not*I or *Pst*I ends, allowing ligation into the appropriate site of GFP in the pCMV/myc/ER/GFP vector. pCMV/myc/ER/GFP was used as a control in transfection experiments, with an inserted stop codon right after GFP.

wtBmp2 was generated by synthesizing cDNA from mRNA extracted from RCS cells using the Qiagen OneStep RT-PCR kit (Qiagen, Valencia, CA) with primers including a *Bam*HI site and a *Xba*I site allowing ligation into pcDNA3.1. This plasmid was used as a template for the production of the Bmp2/GFP fusion construct using a GFP Fusion TOPO TA Expression Kit (Invitrogen Corporation, Carlsbad, CA) according to the manufacturer's instructions.

Bmp2/HA and Bmp2/proHA were constructed utilizing the QuikChange II Site-Directed Mutagenesis Kit (Stratagene, La Jolla, CA) following the directions of the manufacturer. The template employed was wtBmp2 in pcDNA3.1 with primers designed to insert an HA tag at the C-terminus of Bmp2 or two HA tags between amino acids 132 and 133.

The mutated Bmp2 and Bmp2/GFP constructs were made by site-directed mutagenesis, using the QuikChange II Site-Directed Mutagenesis Kit (Stratagene, La Jolla, CA) according to the manufacturer's instructions. For the cleavage site mutant, mtBmp2/GFP, primers were designed to introduce a glycine in place of arginine-279. The ATG1 mtBmp2, ATG1 mtBmp2/GFP, ATG58 mtBmp2, and ATG58 mtBmp2/GFP plasmids were made in the same manner, mutating the A**T**G sites to A**A**G. For the NLSmtBmp2/GFP, the following amino acids were converted to alanines: lysine-275, arginine-276, arginine-286, lysine-287, and arginine-288.

wtBmp4/GFP and wtGDF5/GFP were generated by PCR amplification of Bmp4 from the mouse Bmp4 expression plasmid SP72/BMP4 (provided by Dr. Ronald Koenig at the University of Michigan in Ann Arbor, MI) and GDF5 from the human GDF5 expression vector pCol2a1-CDMP1 (provided by Dr. Yoshihiko Yamada at the National Institute of Dental and Craniofacial Research, Bethesda, MD) and cloned into the GFP Fusion TOPO TA Expression vector as detailed above. The QuikChange II Site-Directed Mutagenesis Kit was again used to generate the mutant constructs. For NLSmtBmp4/GFP the following amino acids were converted to alanines: arginine-288, arginine-289, arginine-302, and lysine-303. Lysine-304 was converted to valine. The ATG1 and ATG83 sites were mutated from A**T**G to A**A**G. For GDF5, the NLSmtGDF5/GFP has the following amino acids changed to alanines: arginine-378, arginine-379, lysine-390, and arginine-391. The ATG1 and ATG173 sites were mutated from A**T**G to A**A**G.

All primers and the complementary oligonucleotide strands were synthesized by Invitrogen Life Technologies. All constructs were verified by DNA sequence analysis in the BYU DNA Sequencing Center, Brigham Young University, Provo, UT.

### Cell culture and transfection

Rat chondrosarcoma (RCS) cells, 10T1/2 cells, and BALB/3T3 cells were maintained in Dulbecco's modified Eagle's medium supplemented with penicillin (50 U/ml), streptomycin (50 μg/ml), L-glutamine (2 mM) and 10% fetal calf serum at 37°C under 5% CO_2_. For transient DNA transfections, *Trans*IT-Jurkat Transfection Reagent (Mirus, Madison WI) was used according to manufacturer's direction.

### Immunofluorescence labeling and microscopy

RCS cells that had been transfected with GFP fusion constructs were fixed using 4% paraformaldehyde/PBS and the nuclei were stained with TO-PRO-3 iodide (Invitrogen Corporation, Carlsbad, CA) according to manufacturer's protocol.

To visualize endogenous Bmp2, Bmp4, and Gdf5, non-transfected RCS, BALB/3T3, and 10T1/2 cells grown on Lab-Tek II Chamber slides (ISC Bioexpress) were fixed in 4% paraformaldehyde, permeabilized and incubated with one of the following primary antibodies: BMP-2 (N-14: sc-6895, Santa Cruz Biotechnology), Bmp4 (JM-5674-100, MBL International), or GDF-5 (N-17: sc-6901, Santa Cruz Biotechnology). After washing, cells were incubated with Alexa Fluor 488-tagged secondary antibodies (Invitrogen Corporation, Carlsbad, CA), mounted in Fluoromount-G (Southern Biotech, Birmingham, AL), and coverslipped. For images of 10T1/2 cells at various stages of the cell cycle, cells were serum-starved for 24 hours. Imaging was performed at different time points after the replacement of 10% fetal calf serum. Antibody specificity was verified by incubating the anti-Bmp2 antibody overnight at 4°C in the presence or absence of a 10-fold molar excess of recombinant human BMP-2 (GenScript, Piscataway, NJ). This pre-absorbed primary antibody was then used to immunostain BALB/3T3 cells as described above.

Cells were imaged using an Olympus IX81 laser confocal microscope with an Olympus UPlanF1 40× 1.3 oil objective and Fluoview version 4.3 image acquisition software, using excitation wavelengths of 488 nm and 633 nm. All imaging was performed in the BYU Confocal Microscope Lab.

### ImageStream^® ^analysis

BALB/3T3 and 10T1/2 cells were trypsinized, washed in phosphate buffered saline (PBS), and fixed in 1% paraformadahyde in PBS for shipping. Nuclei were stained with 4',6-diamidino-2-phenylindole (DAPI) immediately prior to analysis. Analysis of nuclear localization was performed by Amnis Corporation (Seattle, WA) on an ImageStream^® ^multispectral quantitative imaging flow cytometer. Normal single cells expressing GFP were distinguished from untransfected cells, debris, and multicellular aggregates by gating using IDEAS software. Images of individual GFP-expressing cells were analyzed by measuring the 'Similarity' of the GFP and DAPI images. The Similarity score is a log-transformed Pearson's correlation coefficient of the pixel values of the DAPI and GFP images. If GFP is localized to the nucleus, the two images will be similar and have large positive Similarity values [26].

### *In vitro *transcription/translation and *in vitro *digestion assay

Bmp2 protein was synthesized with the incorporation of [^35^S]methionine employing the TNT Coupled Wheat Germ Extract System (Promega, Madison, WI) according to manufacturer's instructions using an expression vector containing the rat Bmp2 cDNA. Ten units of furin (New England BioLabs, Ipswich, MA) or furin plus 2 μM α_1_-PDX (Affinity BioReagents, Golden, CO) were preincubated for 30 min at room temperature. The labeled Bmp2 proprotein was then added, and the reaction was allowed to proceed for 1 hour or 3 hours before products were separated by SDS-PAGE and visualized by autoradiography.

### Immunoblotting

Nuclear and cytoplasmic proteins were separated and isolated using the Cellytic Nuclear Extraction Kit (Sigma, Saint Louis, MO) according to manufacturer's instructions. Western blotting was performed on extracted nuclear and cytoplasmic proteins with the following primary antibodies: anti-HA antibody (EQD Bioscience Inc. San Diego, CA), anti-Bmp2 (Santa Cruz Biotechnology, Inc., Santa Cruz, CA) or anti-Golgi 58K (Sigma, Saint Louis, MO). This was followed by incubation with the appropriate horseradish peroxidase-conjugated secondary antibody.

## Authors' contributions

JEF participated in design of the study, carried out the NLS and start codon mutagenesis, helped with the furin studies, and drafted the manuscript. JLM participated in design of the study, performed the nBmp2/GFP immunohistochemistry experiments and the furin studies, and helped draft the manuscript. TJL performed all the nBmp4 studies, JDJ and DKS performed the nGdf5 studies. SLG documented the localization of endogenous nBmps during mitosis. CAM and JBM initially identified Bmp2 in nuclear extracts from cultured cells. LCB conceived of the study, participated in its design and coordination, and helped draft the manuscript. All authors read and approved the final manuscript.
